# Efficacy and cost-effectiveness of a Transdiagnostic group-based exercise intervention: study protocol for a pragmatic multi-site randomized controlled trial

**DOI:** 10.1186/s12888-021-03541-3

**Published:** 2021-10-30

**Authors:** Sebastian Wolf, Britta Seiffer, Johanna-Marie Zeibig, Jana Welkerling, Leonie Louisa Bauer, Anna Katharina Frei, Thomas Studnitz, Stephanie Rosenstiel, David Victor Fiedler, Florian Helmhold, Andreas Ray, Eva Herzog, Keisuke Takano, Tristan Nakagawa, Saskia Kropp, Sebastian Franke, Stefan Peters, Nadja El-Kurd, Lena Zwanzleitner, Leonie Sundmacher, Ander Ramos-Murguialday, Martin Hautzinger, Gorden Sudeck, Thomas Ehring

**Affiliations:** 1 Faculty of Economics and Social Sciences, Department of Education & Health Research, Institute of Sports Science, University of Tuebingen, Tuebingen, Germany; 2 Faculty of Science, Department of Clinical Psychology and Psychotherapy, Psychological Institute, University of Tuebingen, Tuebingen, Germany; 3 Medical Faculty, Institute of Medical Psychology and Behavioral Neurobiology, University of Tuebingen, Tuebingen, Germany; 4 Department of Psychology, Clinical Psychology and Psychotherapy, LMU Munich, Munich, Germany; 5 Chair of Health Economics, Technical University Munich (TUM), Munich, Germany; 6 German Association for health-related Fitness and Exercise Therapy (German: DVGS), Hürth-Efferen, Germany; 7 AOK Baden-Wuerttemberg, Stuttgart, Germany; 8 Techniker Krankenkasse, Hamburg, Germany

**Keywords:** Exercise, Exercise therapy, Health economics, Outpatient care, Mental health, Mental disorders, Depression, PTSD, Panic disorder, Insomnia

## Abstract

**Background:**

Mental disorders are prevalent and cause considerable burden of disease. Exercise has been shown to be efficacious to treat major depressive disorders, insomnia, panic disorder with and without agoraphobia and post traumatic stress disorder (PTSD).

**Methods:**

This pragmatic, two arm, multi-site randomised controlled trial will evaluate *the efficacy and cost-effectiveness of the* manualized, *group-based six-months exercise intervention “ImPuls”, among physically inactive patients with* major depressive disorders, insomnia, panic disorder, agoraphobia and PTSD *within a naturalistic outpatient context in Germany. A minimum of 375 eligible outpatients from 10 different study sites will be block-*randomized to either ImPuls in addition to treatment as usual (TAU) or TAU only. ImPuls will be conducted by trained exercise therapists and delivered in groups of six patients. The program will combine (a) moderate to vigorous aerobic exercise carried out two-three times a week for at least 30 min with (b) behavior change techniques for sustained exercise behavior change. All outcomes will be assessed pre-treatment, post-treatment (six months after randomization) and at follow-up (12 months after randomization). Primary outcome will be self-reported global symptom severity assessed with the Brief Symptom Inventory (BSI-18). Secondary outcomes will be accelerometry-based moderate to vigorous physical activity, self-reported exercise, disorder-specific symptoms, quality-adjusted life years (QALY) and healthcare costs. Intention-to-treat analyses will be conducted using mixed models. Cost-effectiveness and cost-utility analysis will be conducted using incremental cost-effectiveness and cost-utility ratios.

**Discussion:**

Despite its promising therapeutic effects, exercise programs are currently not provided within the outpatient mental health care system in Germany. This trial will inform service providers and policy makers about the efficacy and cost-effectiveness of the group-based exercise intervention ImPuls within a naturalistic outpatient health care setting. Group-based exercise interventions might provide an option to close the treatment gap within outpatient mental health care settings.

**Trial registration:**

The study was registered in the German Clinical Trials Register (ID: DRKS00024152, 05/02/2021).

**Supplementary Information:**

The online version contains supplementary material available at 10.1186/s12888-021-03541-3.

## Background

Epidemiological data from 2019 suggests that 15.6% of the German population suffered from any mental disorder in 2019 (point prevalence) [1]. Mental disorders result in a considerable burden of disease, for example accounting for 6.4% of overall disability-adjusted life years (DALYS) assessed in the 2019 epidemiological survey [1]. From 2008 to 2018, the proportion of mental disorders among all causes of death increased from 2.2 to 6.1% [2]. The most prevalent disorders in Germany are anxiety disorders and trauma- and stress-related disorders (point prevalence: 7.1%), major depressive disorders (point prevalence: 4.3%), as well as insomnia (point prevalence: 4%) [1, 3]. Notably, these disorders often occur comorbidly [4] and share common underlying aetiological and even maintenance mechanisms, such as high perceived stress [5–7], low self-efficacy [8, 9], sleep disturbances [10], elevated anxiety sensitivity [11–13], or repetitive negative thinking [10, 14].

Worldwide, 27.2% of the DALYS attributable to mental disorders can be explained by major depressive disorders and 16.3% by anxiety disorders [15]. In 2015, health care costs in Germany caused by mental disorders amounted to 44.4 billion euros [16]. Of this, 8.7 billion euros can be attributed to major depressive disorders, 1.7 billion euros to phobic and other anxiety disorders, and 1 billion euros to insomnia. Mental disorders account for 13.1% of total costs and represent the second highest cost group after cardiovascular disorders (46.4 billion euros, 13.7% of total costs). Major depressive (RR = 2.63) and anxiety disorders (RR = 1.41) have also been shown to increase the risk of cardiovascular disease [17]. Besides direct costs (e.g., treatment costs), mental disorders cause indirect costs on the German job market. With 14.4 billion euros overall, mental disorders caused the second-highest lost production costs of all diagnosis groups in 2019 [18]. They further caused 117.2 million days (16.5% of all days) of incapacity to work, which is the longest absences per sick leave of all disorders [19]. Amongst mental disorders, major depressive disorders accounted for the most days of incapacity to work (33.9 million days), followed by trauma- and stress-related disorders (21.6 million days). Anxiety disorders accounted for 7.6 million days and insomnia for 0.5 million days.

Despite the severe negative impact of mental disorders, it is estimated that in Germany only 10% of all affected individuals receive evidence-based treatment and only 2.5% receive psychological treatment [20]. In addition, even those receiving psychological treatment often have to wait before treatment initiation; for example, 40% of outpatients waited three to nine months to start psychotherapeutic treatment in German health care settings [21]. Longer waiting times are associated with worsening and chronicity of symptoms and the development of comorbid conditions [22]. The high prevalence and severe burden of mental disorders in combination with the large gap between people in need for treatment and those actually receiving it [23] illustrates the need to develop alternative efficacious, effective and efficient treatments.

Exercise, defined as physical activity that is planned, structured and repeated, with the primary aim to improve or maintain physical fitness [24], has revealed positive therapeutic effects for diverse mental disorders [25]. Most of the studied exercise interventions include aerobic activities (i.e. running) or a combination of aerobic exercise with strengthening activities. Recent meta-analyses on major depressive disorders and insomnia have shown large effects for exercise that were comparable to those of psychological treatment and psychopharmacological treatment [26, 27]. A recent meta-analysis on PTSD [28] found small to moderate effects; however, in two of the four studies included, the intervention comprised yoga. Looking at more recent evidence from RCTs focusing on interventions including aerobic exercise, large treatment effects were found [29, 30]. For panic disorder (with and without agoraphobia), RCTs have revealed large effects on symptomatology with both, acute exercise and structured multi-week aerobic exercise programs [31, 32]. In addition, moderate to large effects have been reported for exercise as an augmentation to TAU for major depressive disorders, panic disorder and PTSD [30, 33, 34]. Key components of exercise interventions that have shown optimal therapeutic efficacy among patients with major depressive disorders, insomnia, panic disorder with or without agoraphobia and PTSD [35–38] include aerobic exercise at moderate to vigorous intensity (MVAE) either or a combination of MVAE with resistance training, conducted two to three times per week, for 10 weeks with a session duration of at least 30 min, supervised or partially supervised by trained exercise therapists.

Exercise might not only be a promising efficacious treatment but also carry the advantage of being highly efficient, since it can be delivered in group settings with relatively short durations [37], can be offered to patients with heterogeneous and burdensome mental disorders, can be expected to show a low likelihood of adverse effects, comes at a relatively low cost, and is suited to reduce the risk for cardiovascular diseases that frequently occur comorbid with mental disorders [39, 40]. Furthermore, exercise can be performed and continued independently without professional supervision or only remote supervision. However, individuals suffering from mental disorders often have difficulties to initiate and maintain a physically active lifestyle [41], which may be related in part to deficiencies in motivation and exercise-related self-regulatory skills in this population [42]. Reassuringly, there is evidence showing that exercise adoption and maintenance are mediated by motivational and volitional aspects, such as intention strength, action planning and barrier management [43]. A recent meta-analysis shows that especially self-efficacy in building intentions and action planning is crucial for sustained exercise behavior change [44]. One possible way to promote such motivational and volitional aspects are the application of behavior change techniques (BCTs) [45, 46].

Despite the promising evidence for MVAE as an intervention for patients with mental disorders, exercise programs or professional exercise therapy are currently not provided as regular health services within the outpatient mental health care system in Germany. With the aim of combining the current evidence on the efficacy of MVAE and sustained exercise behavior change with specific demands of a real-world outpatient health care setting, ImPuls was developed as a manualized group exercise intervention [47, 48] for physically inactive outpatients suffering from major depressive disorders, insomnia, panic disorder with or without agoraphobia and PTSD. ImPuls integrates recent findings about the optimal modalities of exercise for therapeutic efficacy, such as optimal frequency, intensity, time/duration and type of exercise (FITT criteria) for the targeted disorders and evidence regarding sustainable behavior change by integrating behavior change techniques (BCTs). The components of this intervention are further tailored towards the specific needs of outpatients with mental disorders in the current German mental health care setting. Specific features are 1) the inclusion of a broad range of heterogenous diagnoses for which prior research has demonstrated therapeutic efficacy, 2) intervention delivery in group format, and 3) short duration (i.e., only 4 weeks of supervised MVAE sessions carried out inhouse in each study site). The aim of the current study is to investigate the efficacy and cost-effectiveness of implementing ImPuls within the outpatient mental health care setting in Baden-Württemberg, a representative state in South-West Germany. The following hypotheses will be tested:

1. Participants in the intervention condition, who have received ImPuls in addition to TAU, will show lower global symptom severity at post-treatment and follow-up assessments compared to a control condition with TAU only.

2. Overall costs in the intervention condition will represent a significant saving for the public health system compared to the control condition at post-treatment and follow-up assessments.

3a. The intervention will lead to significantly higher levels of MVAE at post-treatment and follow-up assessments compared to the control condition.

3b. The effect of condition on the reduction of the primary outcome global symptom severity will be mediated by an increase in MVAE.

4. Participants in the intervention condition will show lower disorder-specific symptoms (major depressive disorder, insomnia, panic disorder with or without agoraphobia and PTSD) compared to participants in the control condition at post-treatment and follow-up assessments

We will further assess, if participants in the intervention condition show more instances of clinically significant change compared to participants in the control condition at post-treatment and follow-up assessments (Additional analysis to Hypothesis 1 and 4).

## Methods

### Study design

The study will be led by researchers based at the University of Tuebingen in Germany, and will be conducted in 10 different study sites across Baden-Württemberg, a region in South-West Germany. The entire project will be conducted between September 2020 and February 2024. The study has been registered at the German Clinical Trial Register (ID: DRKS00024152, 05/02/2021) and has been approved by the local ethics committee for medical research at the University of Tuebingen (ID: 888/2020B01, 02/11/2020).

**Fig. 1:**
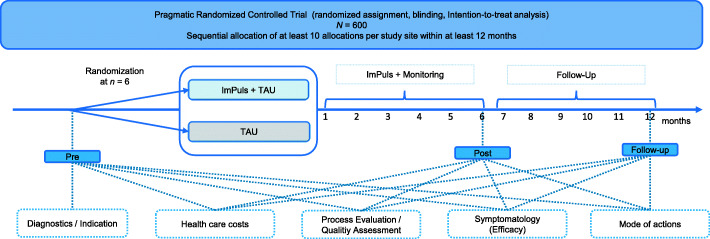
Research design of the pragmatic randomized controlled trial (RCT). Indicated is the random assignment into the intervention and control condition, assessment points and all categories of the outcomes. TAU = treatment as usual, Pre = First assessment prior to randomization, Post = post-assessment 6 months after randomization, Follow-Up = follow-up assessment 12 months after randomization. 6 eligible patients will be block-randomized to either the intervention or control condition

A pragmatic multi-site block-randomized controlled trial with two treatment arms (ImPuls + TAU vs. TAU) and three points of assessment (pre, post, follow-up) will be conducted (see Fig. 1). All outcomes will be included at all assessments. Study completion and reporting will be carried out in accordance with the Consolidated Standards of Reporting Trials (CONSORT), the Template for Intervention Description and Replication (TIDieR) [49] and the Consensus on Exercise Reporting Template (CERT) [50].

### Procedure

Patients will be recruited mainly via inpatient psychiatric departments, family practices, general practitioners and psychiatric and psychotherapeutic outpatient units. The project will be conducted in collaboration with two health insurances, the AOK Baden-Württemberg (AOK) and the Techniker Krankenkasse (TK), who will support the recruitment with a targeted approach of general practitioners, psychiatrists and psychotherapists. All hospitals, clinics and practices will receive information material, such as flyers and posters, to inform their patients about the project. In addition, AOK and TK will publish articles in media distributed for their members during the course of the project. The TK further will inform eligible patients directly via phone calls. Recruitment will be additionally performed via social media posts (Instagram and Facebook), newsletters of professional associations, email distribution lists of universities, local influencers and regional newspapers and television.

**Fig. 2:**
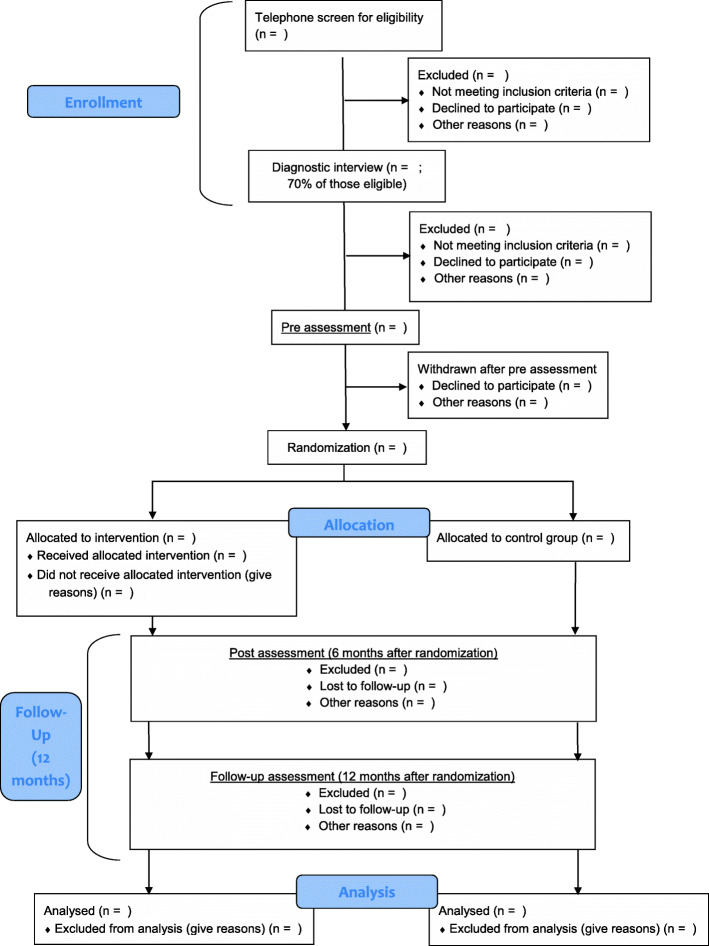
Patient Flow of the pragmatic randomized controlled trial in accordance with CONSORT

Interested patients will first attend a preliminary telephone screening of eligibility criteria and will receive general information about the project (see also Fig. 2). Patients will be screened for somatic contraindications for exercise via the Physical Activity Readiness Questionnaire (PARQ) [51] and will be informed that they have to provide a physician referral for ImPuls before pre-assessment. In case of any suspicion of somatic contraindications, that might oppose participation (e.g., heart diseases or orthopedic problems), patients will be asked to provide an additional medical consult from their general practitioner or a medical specialist. Eligible participants will be invited for a first inhouse meeting taking place in a study site close to their residence. Within the meeting, they will provide written informed consent for study participation, receive information about the study site and will be screened initially for symptomatology related to the exclusion criteria to prepare for the structural diagnostic interview. The telephone screening and initial interview will be performed by trained research assistants. Following this first inhouse meeting, psychologists with a M.Sc. degree undergoing a training in cognitive-behavior therapy, who will be trained by an external expert for structured clinical interviews, will conduct the structured clinical interview for DSM-5 (SKID-5-CV) [52] to confirm eligibility.

Once six patients at the same site will be found to be eligible for participation, they will receive online questionnaires via the web-based data management system REDCap [53, 54] and a accelerometer-based Physical Activity sensors (MOVE 4; movisens GmbH) which will be worn for seven consecutive days (pre-assessment). Online questionnaires and the assessment of physical activity will be carried out within a period of 14 days. On Day 15, the six patients will be randomized as a group to either the intervention or control condition. In case of an assignment into the intervention condition, study sites will have to start the intervention within 14 days. Before the start of the intervention, global symptom as well as disorder-specific symptom severity will be assessed again to check whether participants meet the cut-off criteria for a mental disorder. In each study site, 60 patients are planned to be recruited and randomized, resulting in 10 allocations per site. The intervention group will complete the exercise intervention in addition to TAU, while the control group will receive TAU within the real-world outpatient mental health care setting in Germany. The TAU condition is intended to represent the typical treatment patients receive in the German outpatient health care system. The procedure of the pre-assessment phase will be repeated six months (Post) and 12 months (Follow-up) after randomization. After the completion of all assessments, patients of the control group will receive 450€ as reimbursement for their time.

### Participants

Inclusion criteria are age between 18 and 65 years, membership of the insurance companies AOK BW or TK, fluency in German, no medical contraindications for exercise, and diagnosed according to ICD-10 with at least one of the following disorders: major depressive disorders (F32.1, F32.2, F33.1, F33.2), insomnia (F51.0), panic disorder (F41.0), agoraphobia (F40.0, F40.01) or PTSD (F43.1). Exclusion criteria include: Performing exercise with at least moderate intensity, more than once a week for at least 30 min each, continuously over a period of six weeks within the last three months before study diagnosis, medical contraindication established by the general practitioner or a medical specialist, acute mental and behavioral disorders due to psychotropic substances (F10.0, F10.2-F10.9; F11.0, F11.2-F11.9; F12. 0, F12.2-F12.9; F13.0, F13.2-F13.9; F14.0, F14.2-F14.9; F15.0, F15.2-F15.9; F16.0, F16.2-F16.9; F17.2-F17.9; F18.0, F18.2-F18.9; F19.0, F19.2-F19.9), acute eating disorders (F50); acute bipolar disorder (F31), acute schizophrenia (ICD-10 F20), acute suicidality.

### Sample size

The sample size was determined a priori using power analysis (G*Power, version 3.1.9.2) [55, 56]. Power analysis was conservatively based on the lowest symptom-related post-treatment effect of exercise (vs. TAU/waiting list) on all included clinical disorders, namely the effect size of d = − 0.348 (g = − 0.347) for symptoms of post-traumatic stress disorder [28]. A two-sided *t*-test, alpha level of 0.05, a test power of 80%, an equal cell population, and a dropout rate of 30% were assumed. This calculation resulted in *N* = 375, which is conservative enough to detect the lowest expected treatment effect at the post-treatment phase. However, we regarded this sample size as the minimum and targeted a total sample of *N* = 600, in order to have enough statistical power for further analysis that will be predefined and published in a separate study protocol regarding the evaluation of the entire implementation process based on the Medical Research Council framework [57].

### Randomization

The randomization sequence will be generated independently of the study coordinator and the research team responsible for data collection and management. The sequence will be generated using a varying-size permuted block design, stratified by study site. This procedure will ensure an appropriate balance in the number of treatment and control conditions per study site. Randomization codes will be generated digitally and concealed on a secure system. The group-allocation sequence will be concealed from the research team responsible for data collection and management until the planned unblinding.

### Blinding

No assessors need to be blinded since primary and secondary outcomes will not be based on clinician ratings. Accelerometer-based data will by prepared and processed by trained student assistants according to predefined rules and specifications. Data will be stored and monitored by an external data manager. The sponsor, the research team responsible for data collection and management and any study personnel that stands in direct contact with patients will be blinded regarding the randomization prior to allocation of patients to conditions. In addition, the data analyst will be blinded regarding the treatment allocation. Specifically, he will receive the final dataset, which will be masked for the treatment condition (the “condition” variable only informs Condition A or B but no real labels of the treatment and control conditions). An unblinded data manager will handle the raw data when exporting the data from REDCap.

### Assessments

#### Primary outcome

##### Global symptom severity

Global symptom severity will be assessed by the Global Severity Index (GSI) of the German version of the Brief Symptom Inventory [BSI-18] [58]. The GSI reflects the general mental distress rating on the symptom scales somatization, depression, and anxiety. Each symptom scale consists of 6 items. Thus, 18 items are rated on a 5-point Likert scale (range: 0–4). Higher scores indicate higher distress. Cut-off scores were evaluated separately for men (≥ 10) and women (≥ 13) and have high sensitivity (91.2%) and specificity (92.6%) [59]. Among patients with affective disorders, the GSI has demonstrated good internal consistency (α = .89) and construct validity (*r* = 0.71). Among patients with anxiety disorders the BSI-18 has an internal consistency of Cronbach’s α = .88 and a construct validity of *r* = 0.67 [60].

#### Secondary outcomes

##### Major depressive disorder

The secondary endpoint *depressive symptoms* will be assessed with the PHQ-9 module, assessing symptoms over the last two weeks with nine items, each of them representing one of the DSM-5 (Diagnostic and Statistical Manual of Mental Disorders) criteria for a depressive episode [61, 62]. All items are rated on a 4-point Likert scale (range: 0–3). The sum of all items represents the total score (range: 0–27). Higher scores indicate higher levels of depression. Regarding depressive symptomology, individuals are classified according to the degree of depression severity: absence of depressive disorder (0–4), mild degree of severity (5–10), medium major depression (10–14), severe major depression (15–19) and most severe major depression (20–27). In medical settings the cut-off of ≥10 is used to detect a major depressive disorder [61, 63]. This cut-off was shown to have a sensitivity and specificity of 88 and 85%, respectively [64]. The scale measuring depressive symptoms has an internal consistency of Cronbach’s α = .87 among a representative German sample [61, 65].

##### Insomnia / sleep quality

Nonorganic insomnia will be assessed with the German version of the Insomnia Severity Index [ISI] [66, 67]. The ISI consists of seven items and assesses the severity of sleep onset difficulties, sleep maintenance difficulties, early morning awakening, satisfaction with current sleep, interference with daytime functioning, noticeability of impairment attributed to sleep problems and degree of distress or concern caused by the sleep problem of the past two weeks. The total score ranges from 0 to 28 (range of component scores: 0–3), with a higher score reflecting greater insomnia severity. The cut-off score of ≥11 has shown a high sensitivity (91.4%) and specificity (84.4%) in identifying insomnia. The ISI has shown an internal consistency of Cronbach’s α = .83 among a representative German sample [66].

Sleep Quality will be assessed with the global sleep quality score of the German version of the Pittburgh Sleep Quality Index [PSQI; 68]. The global sleep quality score is the sum of seven sleep component scores (range of component scores: 0–3): subjective sleep quality, sleep latency, sleep duration, habitual sleep efficiency, sleep disturbances, use of sleeping medications, and daytime dysfunction. The global sleep quality score can vary from 0 to 21 with a cut-off score of 5, identifying clinically raised sleep impairment [68]. It has shown a high sensitivity (98.7%) and specificity (84.4%) in identifying insomnia [69].

##### Panic disorder and agoraphobia

The seven-item Generalized Anxiety Disorder scale [GAD-7] [70, 71] assesses symptom severity of generalized anxiety during the last 2 weeks, however shows good performance as a screening measurement for panic disorder and agoraphobia [72, 73] and will therefor serve as a measure for panic agoraphobia symptoms. Items are rated on a four-point Likert scale (range: 0–3). The sum of all items represents the total score (range: 0–21), with scores of ≥5 representing mild, scores of ≥10 moderate and scores of ≥15 severe anxiety symptom levels, respectively. The cut-off score of ≥10 has shown high sensitivity (89%) and specificity (82%) [71]. Among a representative German sample, the GAD-7 has an internal consistency of Cronbach’s α = .85 [74].

Besides the GAD-7, symptoms of panic disorder and agoraphobia symptoms will be assessed with the three-items subscale panic of the 6-items scale anxiety of the BSI-18 [58]. Current evidence suggests a four-factor structure of the BSI-18 that retains the somatization and depression symptom scales but splits the anxiety symptom scale in two factors: General anxiety and panic [75, 76]. The subscale panic of the BSI-18 consists of three items that are rated on a five-point Likert scale (range: 0–4). Among a German outpatient sample that was surveyed five times after the 2nd, 6th, 10th, 18th and 26th therapy session the best fitting model according to Akaike Information Criterion (AIC) was always the model with four factors, compared to one- and three-dimensional models [75]. Among patients with anxiety disorders, the GSI of the symptom scale anxiety has demonstrated good internal consistency (α = .83) and construct validity (*r* = 0.67) [60].

##### Posttraumatic stress disorder

To assess symptoms of PTSD, the German version of the PTSD Checklist for DSM-5 (PCL-5) [77] will be used. The questionnaire is a self-report measure that consists of 20 items corresponding to the DSM-5 criteria for PTSD. Participants report their intensity of symptoms over the past four weeks on a five-point Likert scale (0 = not at all to 4 = extremely; total range 0–80). Higher scores indicate higher levels of PTSD. The German Version shows high internal consistency (α = .95), high test-retest reliability (*r* = .91) and a high construct validity (*r* = .77). A cut-off of 33 indicates clinically relevant symptomatology.

##### Health related quality of life

Health related quality of life will be assessed by the German version of the EQ-5D-5L questionnaire [78, 79]. It consists of five items concerning the domains mobility, self-care, usual activities, pain or discomfort and anxiety or depression with five answer alternatives each (range: 1–5). The combinations of the answer alternatives can be described with a five-digit number (i.e. the pattern 11,111 indicates the optimal health state). Through the EQ-5D-5L questionnaire Quality Adjusted Life Years (QALY) are captured for the economic evaluation. The EQ-5D-5L has an internal consistency of Cronbach’s α = .86 among German chronic heart failure patients. Current data concerning internal consistency in patients with mental disorders exists for the Spanish version of the EQ-5D-5L. Among Spanish patients with major depression the EQ-5D-5L has an internal consistency of Cronbach’s α = .77 [80].

##### Routine data of the health insurances/health care costs

For the economic evaluation, patients’ routine data collected 6 months before the intervention, during the time of the intervention, and six months after the intervention will be provided by the two participating statutory health insurers (AOK and TK). This data will include patients’ master data, such as gender and age, as well as patient treatment costs. Parameters for treatment costs will comprise costs of inpatient and outpatient care as well as medication, medicals aids or days of incapacity to work. Routine data for each patient will be provided for the time of the intervention as well as one year prior and one year after. The relevant costs are assessed and aggregated as quantities. In addition, cost parameters resulting from the intervention and the implementation will be considered. Subsequently, routine insurance data will be linked to primary data collected.

##### Exercise behavior/MVAE

The assessment of self-reported exercise duration and frequency and the assessment of accelerometer -based moderate to vigorous physical activity will serve as two proxies for MVAE. Exercise in minutes per week will be assessed using the self-report Exercise Activity Index of the Physical Activity, Exercise, and Sport Questionnaire ([BSA questionnaire [81];). Participants specify type, duration, and frequency of exercise in the last four weeks. Moderate to vigorous physical activity (MVPA) will be assessed via accelerometer-based sensors (Move 4, movisens GmbH). The sensor assesses physical activity of a person based on kinematic data in three dimensions and atmospheric air pressure. This allows to estimate the amount of physical activities of different intensities for a specified time period based on validated algorithms [82]. Patients will wear the sensors for seven consecutive days. Volume of moderate to vigorous physical activity will be indicated in minutes/week and calculated by the daily physical activity of at least moderate intensity (≥ 3 MET)/minutes.

#### Assessments to ensure internal validity

Symptom severity at randomizationSymptom severity might change or fluctuate from the diagnostic interview or pre-assessment to the start of the intervention. In order to ensure clinically relevant transdiagnostic and disorder-specific symptomatology with the start of the intervention, scales that assess all primary and secondary outcomes will be presented again between randomization and group start: BSI-18 [58], ISI [66, 67], PSQI [68], PHQ-9 [61, 62], GAD-7 [70, 71], PCL-5 [77].

(Serious) Adverse EventsAdverse events (AE) will be assessed at pre, post-1, and follow-up assessment. AEs and Serious Adverse events (SAEs) can be further reported by patients or therapists at a central phone number. AEs and SAEs will be documented and SAEs will be reported to an independent Data Safety and Monitoring Board, which will discuss adjustments to or discontinuation of the entire study.

**Fig. 3:**
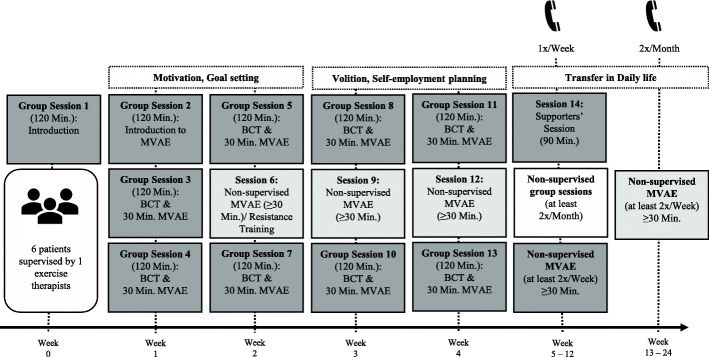
The temporal program structure and content overview of ImPuls. The dark gray boxes illustrate the supervised sessions with group meetings (“Group Session”) and moderate to vigorous aerobic exercise (“MVAE”) as well as the supporters’ session in week 5. The group sessions integrate different behavioral change techniques (“BCT”) to enhance motivational and volitional skills with the long-term aim for maintenance aerobic exercise. The medium gray boxes illustrate non-supervised aerobic exercise in which the patients can choose independently any aerobic exercise that best fits to their interests and needs. The light gray box illustrates the non-supervised group sessions from week 5 to 24 in which patients complete the aerobic exercise together but without the therapist. The telephones cartoons represent telephone contacts during the non-supervised time to monitor the long-term maintenance of aerobic exercise. The entire program is supported by the ImPuls smartphone application, developed especially for ImPuls

Treatment Fidelity (adherence to protocol of study therapists)Core elements of the manualized ImPuls intervention have been determined a priori. All inhouse sessions (see Fig. 3 and Table 1) of all therapists at all study sites will be video-taped despite outdoor MVAE. External research assistants will be trained to rate fidelity based on deliverance as intended in the manual. A treatment fidelity score will be determined as a mean of all ratings. 10% of all video-taped sessions will be randomly selected and analyzed. A fidelity score of ≥90% is assumed.

**Table 1:** Overview of behaviour change techniques included in ImPuls

Focus	Technique
Motivational (mainly weeks 1–2)	Education about positive and negative effects of exercise
	Education about optimal modalities of exercise to experience positive psychological effects
	Selection of a preferred activity and level of intensity Self-monitoring of exercise Imagination of goals being reached in the future
	Goal setting
	Self-monitoring of goal achievement
	Reflection about positive experiences/effects with/of exercise Reflection about self-monitoring of exercise
Volitional (mainly weeks 3–4)	Identification of barriers to exercise
	Techniques to overcome barriers
	Exercise planning through training plans
Motivational and volitional (weeks 4–24)	Social support (family, friends) through the supporters meeting Social support (other patients, self-organized group meetings)
	Self-monitoring of goal achievement
	Exercise self-monitoring trough diaries, training plans and analysis of FITT criteria (optimal modality)

All techniques/approaches will be delivered via the exercise therapists and protocoled, supported and guided by the ImPuls App

##### Dropout and attendance rates (patients)

A drop-out rate less than 30% and attendance rate ≥ 80% is assumed. Attendance within the supervised (weeks 0–4; please refer to Fig. 3) and partially supervised (weeks 5–24) period will be assessed via the ImPuls smartphone application and attendance lists by the exercise therapists/study site. Participants who miss more than 4 consecutive sessions during the four weeks of the supervised phase (of weeks 0–4) of the intervention (≥40%, due to any reason) but continue the study assessments are defined as treatment-dropout, since they did not receive the intended dose of the intervention. They can still participate in the remaining sessions of the intervention and will be asked to complete all remaining assessments. Participants who miss more than 4 consecutive sessions during the four weeks of the supervised phase (of weeks 0–4) of the intervention (≥40%, due to any reason) and discontinue the study assessments will be defined as study dropout. Participants who will discontinue the treatment as well as the assessments will be defined as study dropout. All participants agree to be asked to specify their reasons for study/treatment-dropout on a voluntary basis (once, after treatment/study dropout). If available, reasons for discontinuation will be reported. If more than 50% of participants will discontinue their participation in the intervention group during the first five weeks (weeks 0–4) of the delivery, discontinuation of the group due to lack of economic efficiency for the study sites is possible. If only one participant of the intervention group remains, he or she can no longer receive the intervention, since delivery of the intervention to less than two participants no longer qualifies as “group-based intervention”.

##### MVAE dose within the supervised period (patients)

Frequency (at least twice a week), intensity (mean intensity of least 64% of maximum heart rate) and duration (at least 30 min of MVAE in each session) of exercise during the supervised period (weeks 1–4) will be assessed via the ImPuls smartphone application.

##### Expectations, motivation and satisfaction

Validated scales adapted for use in the context of ImPuls will be employed to assess patients’ outcome expectations [83], motivation [84] and satisfaction with the intervention [85], as well as exercise therapists’ motivation [86], and satisfaction with the intervention [87]. On all scales, mean scores falling within the upper quartile will be judged as indicative of high motivation for and acceptability of treatment, respectively, in patients and exercise therapists.

##### Process evaluation

In addition to the goals related to investigating efficacy and cost-effectiveness of ImPuls reported in this study protocol, the overall project pursues additional goals focusing on process evaluation and implementation of ImPuls based on the Medical Research Council Guidance [57] in the routine health care system, which will be described in a separate study protocol.

#### Intervention

##### Treatment as usual (TAU)

The TAU condition will be modelled to represent the typical treatment patients receive in the German outpatient health care system. Therefore, patients will not be actively provided with any treatment but patients are allowed to receive any intervention that is available to them. Any evidence-based treatment provided by the outpatient mental health care system will be recorded, i.e., any psychiatric/pharmacological or psychological/psychotherapeutic intervention. Interventions, delivery or dosage of the intervention can be changed and adapted during the course of the study.

##### ImPuls

The exercise intervention “ImPuls” [88] will be delivered to groups consisting of 6 patients and will be divided into a supervised and partially-supervised period. BCTs, such as goal setting, self-monitoring, formation of concrete exercise plans and coping planning, will be integrated to promote sustained exercise behaviour change [45, 46, 89]. The intervention structure and contents are displayed in Fig. 3 and Table 1. Please also refer to the TIDieR-Checklist (Additional file 1_TIDieR-Checklist.pdf as supplementary material). Participants will receive ImPuls in addition to TAU.

##### Supervised period (weeks 0–4)

Patients will participate in a combination of supervised MVAE sessions and group sessions with educative elements integrating BCTs (see Table 1) in groups with a total duration of 120 min each session. Supervised MVAE will be provided twice a week and will consist of either running or fast walking. MVAE will last 30 min and participants can choose between a standardized interval-based or endurance method protocol. Both training methods will be conducted with at least moderate intensity, which is tracked by a heart rate monitor (SIGMA iD.FREE) combined with a chest strap (SIGMA R1 Bluetooth Duo Comfortex+) and the Borg Rating of Perceived Exertion (RPE) Scale [90]. Moderate to vigorous intensity is defined as at least 64% of maximum heart rate, subtracting age from 220 [91] and at least 13 points of the RPE Scale [90]. The ImPuls smartphone application (“ImPuls-App”) developed specifically for ImPuls supports the participants and therapists during MVAE. In weeks 2, 3 and 4 patients will engage in additional 30-min MVAE, which is chosen based on their own interests and preferences. Therapists provide a list of MVAE highlights in each study site (i.e. offers in local sport clubs, yoga studio, gyms) which can be found and selected in the “ImPuls-App”.

##### Partially supervised period (weeks 5–24

Participants will be asked to engage in 30-min non-supervised MVAE at least twice a week. Regular MVAE will be planned through specific training schedules and accompanied by activity diaries, self-monitoring of goals and volitional strategies and weekly (weeks 5–12)/biweekly (weeks 13–24) phone calls with the exercise therapist, intending to maintain motivation, volition, and adherence to exercise. Training plans and all documentations will be executed and coordinated via the “ImPuls-App”. Information will be shared with the therapists in advance prior to the phone calls. A session for patient’s supporters (e.g., friends, partner) will be scheduled in Week 5 to inform them about the possibilities to support the participants in transforming their intentions into action.

##### ImPuls smartphone application (“ImPuls-app”)

The ImPuls App will support the participants with options for exercise planning (training plans), exercise guidance (interval training, resistance program, ratings of perceived exertion), self-monitoring of goal achievements (analysis and feedback of FITT criteria and goal achievements), mitigation of barriers and a knowledge base. A web-based application for exercise therapists will support planning and logging individual and group sessions with clients (calendar, attendance, active participation, notes). Furthermore, the participants can share some or all of their data (such as their exercise schedule or their plans for overcoming barriers) with their exercise therapists via the secure channels between the ImPuls App and the web-based platform. This will enable direct feedback of therapists to their clients. Both Apps run on Google Android and on Apple iOS. All data generated in the apps will be protected by encryption on the smartphone. All communication between smartphones (ImPuls App), browsers (Therapist’s App) and the central server will be protected by established encryption protocols, too. All components in Table 1 will be documented in or provided by the ImPuls App.

##### Study therapists/exercise therapists

To carry out the intervention, exercise therapists will be required to have one of the following academic or comparable basic qualifications as physical activity and exercise professionals with a training period of at least 3 years: academic degree in exercise or movement science with at least 10 ECTS e.g.(i.e., 250 h) practice and 20 ECTS (i.e., 500 h) theory (e.g. Magister, Bachelor, Master, Diploma Physical Education, Exercise Science, Exercise Physiology), non-academic technical college degree Exercise and Caring/Therapeutic Gymnastics with at least 21 semester hours per week, non-academic technical college degree „Physical Educator as liberal profession“, academic and non-academic degrees in Physiotherapy. Moreover, a specific additional therapeutic qualification DVGS e.V. with 5 ECTS (i.e. 150 h) overall is required with the following content: Physical Activity-related Health Competence (2 ECTS, i.e. 100 h), Basics in Health Science and Health Pedagogy (1 ECTS; i.e. 50 h), Basics in Psychiatry, Psychosomatics and Addiction (1 ECTS, i.e. 50 h), Affective Disorders (1 ECTS, i.e. 50 h).

All exercise therapists will be trained in ImPuls and will receive a two-day and a one-day training at the University of Tuebingen. In case of missing a part of the training, therapists will be offered an individual training to catch up the missed elements. After the start of the first intervention at each study site, exercise therapists will receive a specific inhouse training within the first two weeks to discuss and solve problems and questions that arise with intervention start. After the inhouse training, exercise therapists will be offered monthly online-supervisions by two external and independent supervisors, one with a background in cognitive-behavioral therapy (CBT) and one with a background in exercise therapy. Supervision can be attended on a voluntary basis.

##### Study sites

Study sites are local outpatient rehabilitative and medical care facilities which were selected to cover the different regions in Baden-Württemberg. They are required to have premises for the group discussions (a room for patient education, more than 12 square meters) and the supporters’ session (a suitable room for 10–20 persons; more than 30 square meters). An outdoor area for endurance-oriented exercise has to be available and emergency equipment must be on-hand. Every study site needs to have two to three employed exercise therapists with the described specific qualifications. Study sites enter into contracts with the German Association for health-related fitness and exercise therapy (DVGS e.V.) to ensure compliance to the above-mentioned structural requirements and reimbursement for conducting ImPuls.

#### Statistical methods

To test Hypotheses 1, 2, 3a and 4 we will use multilevel modeling to establish the treatment effects on the primary outcome (global symptom severity) and the secondary outcomes (major depressive disorders, insomnia, panic disorder, agoraphobia, PTSD, QALYs, exercise). In these analyses, each outcome will be predicted by the group (ImPuls + TAU vs. TAU), time (pre vs. post vs. follow-up), and their interaction. Given that the randomization is stratified by study site, we will account for the effects of study site in all analyses. All analyses will be performed on intention-to-treatment basis, using maximum likelihood estimations.

To test Hypothesis 3b, mediation analyses will be performed to test the indirect effects of the treatment on the primary outcome that are mediated by the changes in the putative mediators (self-reported exercise in minutes per week, accelerometry-based moderate to vigorous physical activity (MVPA); from pre to post, pre to follow-up and post to follow-up). We will compute standardized change scores for the outcome and mediators [92]. In a path model (specified in the framework of structural equation modeling), the group variable predicts the changes in the mediators, and these mediating factors further predict the change score in the outcome. The indirect effects are defined by the products of the group-mediator and mediator-outcome effects. As exploratory analyses, we will also examine other forms of mediations proposed by Goldsmith et al. [93], encompassing cross-lagged effects and latent change score models. As recommended by Usami et al. [94], we will first test whether these models fit the data well (and which model fits the data best), and then investigate the indirect effects of the treatment on the outcome.

To further analyze clinically significant changes (additional analysis for Hypothesis 4 and 5), the Reliable Change Index (RCI) [95–97] will be computed for each individual between pre-, post and follow-up assessments. Jacobson and Truax suggest combining the RCI with an estimation of a cut-off point between a functional (nonpatient) and dysfunctional (patient) population, describing a clinically significant change [96, 98]. We will check if individual scores are more than 2 SD away from the mean of the complete sample. Each individual will be classified into one of four categories: recovered (individual has passed cut-off point of clinically significant change and the RCI is greater than 1.96), improved (individual has not passed cut-off point but the RCI is greater than 1.96), unchanged (individual has not passed neither cut-off point nor is the RCI greater than 1.96), or deteriorated (the individual’s RCI is greater than − 1.96). Non-parametric generalized mixed models will be conducted to analyze differences between intervention and control group.

Missing data will be handled by multiple imputations.

#### Economic evaluation

To test Hypothesis 2, the economic evaluation will determine the cost-effectiveness and cost-utility of the intervention condition comparing the intervention condition with the control condition. Results are illustrated as incremental cost-effectiveness and cost-utility ratios, which illustrate the additional costs [Euro] incurred in relation to the additional unit of effectiveness [transdiagnostic symptomatology] or utility [QALY]. Quality Adjusted Life Years (QALY) are captured by using the EQ-5D-5L questionnaire.

The statistical uncertainty of the incremental cost-effectiveness ratios will be evaluated by applying cost-effectiveness acceptability curves (CEAC). During the intervention period and follow up time, costs of outpatient and inpatient care, outpatient medication and days of incapacity to work are displayed as quantities. In addition, cost parameters resulting from the intervention and the implementation are considered.

The perspective of the statutory health insurances is adopted and the results are examined with regard to a transferability to the statutory health insurance populations. Sensitivity analyses, additional explorative subgroup analyses and statistical uncertainty analyses will provide a differentiated illustration of the results of the economic evaluation. Differences in costs related to disparities in age or comorbidities are additionally analyzed using mixed models.

## Discussion

Exercise therapy is currently not provided within the outpatient mental health care system in Germany, despite the promising effects of exercise found for different mental disorders [25], and despite its promise in bridging the severe gap of people in need and people actually receiving evidence-based treatment. Especially for major depressive disorders, insomnia, panic disorder with or without agoraphobia and PTSD, there is evidence suggesting that it is an efficacious treatment [26–34]. The group-based exercise intervention ImPuls integrates recent findings about optimal modalities of exercise for therapeutic efficacy for the above-mentioned disorders and sustainable behavior change by integrating behavior change techniques (BCTs). The components of this intervention are further tailored towards the specific needs of outpatients with mental disorders in the current German mental health care setting: 1) including a broad range of heterogenous diagnoses for which prior research has demonstrated therapeutic efficacy, 2) conducting the intervention in a group format of 6 patients supervised by 1 exercise therapist, 3) short duration (i.e., only 12 supervised inhouse sessions), 4) integration of a smartphone application facilitating the transition to self-dependent exercise, empowering the patient and the therapist.

A recent pilot study evaluating the efficacy of ImPuls within a monocentric randomized controlled trial for patients with major depressive disorders, insomnia, anxiety disorders, PTSD and ADHD showed therapeutic effects comparable to disorder-specific exercise interventions or established treatments, such as CBT [48]. Besides the promising efficacy, the intervention also showed a low drop-out rate (18%) and a large and long-lasting increase of exercise behavior in the intervention group. To the best of our knowledge, this large pragmatic multi-site trial will be the first in evaluating the efficacy and cost-effectiveness of a group exercise program within a naturalistic outpatient mental health care setting among patients with heterogeneous disorders. If results are promising, exercise might be seen as an efficacious, effective, efficient and easily accessible treatment that might be an option to improve the treatment gap within the outpatient mental health care setting in Germany.

Specific challenges might arise during the current trial. First, recruitment success might depend on regional circumstances. Some study sites are located in big cities and some in smaller districts with smaller populations. Thus, participation demands might be higher in some study sites compared to others, which could lead to unbalanced participation rates between sites or might have further effects on the motivation of study therapists since some might wait a long time to start their first group while others might be pressured to start the intervention. However standardized training and adaptations in recruitment procedures might counteract these challenges. The effects of study sites will be considered in the statistical models. Second, recruitment and implementation started in March 2021 during the third wave of the Covid-19 pandemic in Germany. Thus, patients might be hesitant or anxious to participate in the study. There might be a bias due to a potentially low participation rates of patients with anxiety disorders. This possible selection bias will be also modelled statistically.

In addition, another study protocol will be published that goes beyond this study protocol and addresses in more detail the relevant outcomes of the implementation process (implementation, context, mechanism of impact), following the MRC framework [57].

## Supplementary Information

**Additional file 1.** TIDieR-Checklist.pdf (TIDieR Checklist).

## Data Availability

Individual participant data that underlie the results reported in this article will be published after deidentification. Documents that will be shared further: Study protocol, statistical analysis plan, analytic code, aggregated individual study data. Routine/administrative data from health insurances will not be made available. Access to data will be provided for anyone legitimately interested in it. Analytic code and aggregated individual study data will be made available on an online repository immediately after publication (or within the peer review process). Participants give informed consent to publish their data after deidentification (despite the routine/administrative data from the health insurances).

## Abbreviations

AE: Adverse events
AOK: Allgemeine Ortskasse (German health insurance)
App: (smartphone) Application
BCT: Behavioral change techniques
BSA: Physical Activity, Exercise, and Sport Questionnaire
BSA questionnaire: Physical Activity, Exercise, and Sport Questionnaire
BSI-18: Brief Symptom Inventory 18
CBT: Cognitive behavioral therapy
CEAC: Cost-effectiveness acceptability curves
CERT: Consensus on Exercise Reporting Template
CONSORT: Consolidated Standards of Reporting Trials
DALY: Disability adjusted life years
DSM-5: Diagnostic and Statistical Manual of Mental Disorders (5th version)
DVGS: German Association for health-related fitness and exercise therapy
ECTS: European Credit Transfer and Accumulation System
EQ-5D-5L: Standardized measure of health-related quality of life
FITT: Frequency, intensity, time, type
GAD-7: Generalized Anxiety Disorder scale
ICD-10: International Classification of Diseases (version 10)
ISI: Insomnia Severity Index
ITT: Intention-to-treat
MET: Metabolic equivalent of task
MRC: Medical Research Council Guidance
MVAE: Moderate to vigorous aerobic exercise
MVPA: Moderate to vigorous physical activity
PARQ: Physical Activity Readiness Questionnaire
PSQI: Pittsburgh Sleep Quality Index
PSQI: Pittburgh Sleep Quality Index
PTSD: Posttraumatic stress disorder
QALY: Quality-adjusted life years
RCI: Reliable change index
RCT: Randomized controlled trial
REDCap: Research Electronic Data Capture (software)
RPE: Rating of Perceived Exertion
RPE Scale: Rating of Perceived Exertion Scale
SAE: Serious adverse events
SKID-5-CV: Structured clinical interview for DSM 5 – clinical version
TAU: Treatment as usual
TIDieR: Template for Intervention Description and Replication
TK: Techniker Krankenkasse (German health insurance)
